# Your Cheatin’ Voice Will Tell on You: Detection of Past Infidelity from Voice

**DOI:** 10.1177/1474704917711513

**Published:** 2017-06-04

**Authors:** Susan M. Hughes, Marissa A. Harrison

**Affiliations:** 1 Psychology Department, Albright College, Reading, PA, USA; 2 Psychology Program, Penn State Harrisburg, Middletown, PA, USA

**Keywords:** voice attractiveness, infidelity detection, voice pitch, cheating

## Abstract

Evidence suggests that many physical, behavioral, and trait qualities can be detected solely from the sound of a person’s voice, irrespective of the semantic information conveyed through speech. This study examined whether raters could accurately assess the likelihood that a person has cheated on committed, romantic partners simply by hearing the speaker’s voice. Independent raters heard voice samples of individuals who self-reported that they either cheated or had never cheated on their romantic partners. To control for aspects that may clue a listener to the speaker’s mate value, we used voice samples that did not differ between these groups for voice attractiveness, age, voice pitch, and other acoustic measures. We found that participants indeed rated the voices of those who had a history of cheating as more likely to cheat. Male speakers were given higher ratings for cheating, while female raters were more likely to ascribe the likelihood to cheat to speakers. Additionally, we manipulated the pitch of the voice samples, and for both sexes, the lower pitched versions were consistently rated to be from those who were more likely to have cheated. Regardless of the pitch manipulation, speakers were able to assess actual history of infidelity; the one exception was that men’s accuracy decreased when judging women whose voices were lowered. These findings expand upon the idea that the human voice may be of value as a cheater detection tool and very thin slices of vocal information are all that is needed to make certain assessments about others.

The sound of the human voice can communicate a wealth of information to listeners that is irrespective of semantic content. Evidence suggests that there are cues in the human voice that can reveal accurate information about a speaker’s sex (Lass, Hughes, Bowyer, Waters, & Bourne, 1976; Lass, Tecca, Mancuso, & Black, 1979), age (Hughes & Rhodes, 2010; Krauss, Freyberg, & Morsella, 2002), race (Lass et al., 1979; Walton & Orlikoff, 1994), height and weight (Krauss et al., 2002; Lass & Colt, 1980; Lass & Davis, 1976), body size and configuration (Hughes, Harrison, & Gallup, 2009; Pisanski et al., 2015), social status (Brown & Lambert, 1976), personality traits (Addington, 1968; Zuckerman, Hodgins, & Miyake, 1990), and emotional and mental state attributes related to deception (Ekman, Friesen, & Scherer, 1976; Streeter, Krauss, Geller, Olson, & Apple, 1977). Voice attractiveness has been linked to greater bilateral body symmetry (Hughes, Harrison, & Gallup, 2002; Hughes, Pastizzo, & Gallup, 2008), greater facial symmetry (Abend, Pflüger, Koppensteiner, Coquerelle, & Grammer, 2015; Hill et al., 2017), lower waist-to-hip ratio in women and higher shoulder-to-hip ratio in men (Hughes, Dispenza, & Gallup, 2004), and to the fertile phase of a woman’s menstrual cycle (Pipitone & Gallup, 2008). Additionally, subjects can accurately match voice samples to a target’s facial photograph over 75% of the time (Krauss et al., 2002). Voice is also used in clinical assessment for identifying certain neurological disorders such as motor neuron disease, conditions affecting the basal ganglia (Gamboa, Jimenez-Jimenez, Mate, & Cobeta, 2001), and for monitoring psychoactive drug effects (Scherer & Zei, 1988).

In particular, voices relay important information pertaining to mating success and sexual behavior. For instance, Apicella, Feinberg, and Marlowe (2007) found that men with lower voice pitch have higher reproductive success (i.e., fathered more children). Likewise, Atkinson et al. (2012) found a direct relationship between female fundamental frequency and several measures of genetic fitness among a group of indigenous women. There is also evidence showing that both sexes with voices rated as sounding more attractive had first sexual intercourse at an earlier age, a greater number of sexual partners, a greater number of extra-pair copulation (EPC) partners, and a higher number of partners whom they had intercourse with who were involved in another relationship (i.e., were themselves chosen as an EPC partner; Hughes et al., 2004).

It appears that listeners are unconsciously tuning into voice pitch to ascribe infidelity risk to speakers. O’Connor, Re, and Feinberg (2011) manipulated the pitch of men’s and women’s voices to test whether raters would make cheating ascriptions based on voice pitch. Using a forced-choice paradigm, they found that women, but not men, rated men with lower pitched (masculinized) voices as more likely to cheat. In contrast, men, but not women, rated women with higher pitched (feminized) voices as more likely to cheat. O’Connor et al. (2011) attributed these results to listeners cuing in to the hormonal profiles typically associated with opposite-sex infidelity risk. That is, testosterone is associated with a lower pitched voice in men (Dabbs & Mallinger, 1999; Evans, Neave, Wakelin, & Hamilton, 2008), and it has been positively associated with number of sex partners (Bogaert & Fisher, 1995). Indeed, it has been shown that men’s pitch was negatively correlated with their reported number of sexual partners over the past year (Puts, 2005).

On the other hand, for women, estrogen is associated with higher pitched voices (Abitbol, Abitbol, & Abitbol, 1999) and has been positively associated with a greater likelihood to engage in flirting, kissing, or having serious affairs with someone other than a primary partner (Durante & Li, 2009). Further, women generally find lower pitched male voices as more attractive and prefer deeper voices to higher ones (Apicella, Feinberg, & Marlowe, 2007; Collins, 2000; Feinberg, Jones, Little, Burt, & Perrett, 2005; Karpf, 2006; Riding, Lonsdale, & Brown, 2006; Re, O’Connor, Bennett, & Feinberg, 2012), especially during the most fertile time of the menstrual cycle (Puts, 2005). Men assess higher pitched female voices as sounding more attractive (Collins & Missing, 2003; Feinberg, DeBruine, Jones, & Perrett, 2008; Re et al., 2012), particularly in short-term mating contexts (Puts, Barndt, Welling, Dawood, & Burriss, 2011). However, other studies have shown that when females lowered the pitch of their voice, men rated their voices as sounding “sexier” (Hughes, Farley, & Rhodes, 2010; Hughes, Mogilski, & Harrison, 2014). Similarly, Babel, McGuire, and King (2014) also found that female voices with slightly lower than average fundamental frequency (i.e., pitch) were rated as more attractive by listeners. When Puts and colleagues (2016) examined a large sample of female voices and statistically controlled for several other acoustic parameters, they found that pitch did not predict women’s attractiveness for either short- or long-term relationships. Despite the contradictory literature of what constitutes an attractive female voice, listeners may simply have assumed that those with more attractive voices were more likely to cheat simply due to increased sexual opportunity.

These findings raise the questions of whether voice can be used to determine whether another has engaged in sexual infidelity in actuality, and if this is possible, whether vocal signals of high mate value (e.g., voice attractiveness and attractive pitch) are the only determinants used to assess if a speaker has a history of infidelity. There may be subtle signals in the human voice and/or the way in which a person speaks that can lead to accurate perceptions of a speaker’s history of infidelity. This idea is in line with Ambady and Rosenthal’s (1992) “thin slice” theory that posits people can make reasonably accurate assessments of others based on very minimal observations. As an example, Rhodes, Morley, and Simmons (2013) showed that women could accurately judge an unfamiliar man’s past unfaithfulness from minimal facial cues, and women used masculinity as the valid cue to do so. People are also able to identify infidelity in romantic relationships simply from brief observations of behavior. Lambert, Mulder, and Fincham (2014) found that participants could accurately detect which targets had cheated on their romantic partners by watching a short video clip of the pair interacting. Lambert and colleagues determined that this detection was mediated by ascriptions of commitment and trustworthiness of the persons they were observing.

Research in evolutionary psychology has also routinely supported this thin slice notion in the arena of vocal research (i.e., very brief voice samples can be used to predict a myriad of physical and behavioral elements). For instance, Hughes, Harrison, and Gallup (2009) showed that listeners can select a speaker’s body configuration at a degree greater than chance from only hearing their voice. Specifically, listeners were able to match a man’s voice with a silhouette approximating his shoulder-to-hip ratio, and listeners were able to match a woman’s voice with a silhouette approximating her waist-to-hip ratio. This ability may be important in mate selection, as these ideal sex-specific body configurations are a result of hormonal profiles signaling mate quality (see Hughes et al., 2009).

## Infidelity Detection

Aside from using vocal cues, there seem to be other features that individuals use for infidelity detection. Shackelford and Buss (1997) identified a long list of specific behavioral cues that can lead people to suspect a partner’s infidelity (whether they were real or alleged) and included among them were observations of partner engaging in changes in their normal routines; exaggerated affection; a sudden increase in sexual interest; emotional disengagement; passive rejection; being overly critical, argumentative, or apathetic; reluctance to spend time with one’s partner; and reluctance to discuss a particular individual.

The proclivity to cheat may also be revealed through personality traits. As examined across 10 world nations, high extroversion strongly predicts infidelity (Nettle, 2005; Nettle & Clegg, 2008) while low agreeableness and conscientiousness is associated with infidelity (Schmitt, 2004). Therefore, it can be argued that choosing a long-term mate with such traits may expose oneself to a higher risk of infidelity.

From an evolutionary standpoint, it would be reproductively advantageous for both men and women to assess accurately a partner’s proclivity to cheat, especially considering that acts of infidelity occur at high rates. Wiederman and Hurd (1999) found that 75% of men and 68% of women admitted to engaging in extradyadic (ED) dating activity and/or some form of ED sexual behavior (e.g., kissing, fondling, intercourse) while involved in a committed, romantic relationship. For a man, it is adaptive to be able to detect signs of infidelity because of the possibility of cuckoldry and investing in offspring who are not genetically related. For a woman, it is adaptive to be able to detect signs of infidelity so as to avoid diversion of resources by her mate and/or avoid abandonment, which would have left a woman and her offspring vulnerable in the ancestral environment (Buss, 1995; Buss & Shackelford, 1997). Thus, both men and women have different but reproductively necessary reasons for detecting cheaters. Because of the interpersonal and reproductive costs that men and women can incur from a partner’s EPC (Shackelford, 1997), humans have likely evolved strategies of detecting individuals that have an increased likelihood of being unfaithful.

Presumably, because the high cost of failing to detect a partner’s infidelity places men at risk for cuckoldry, men’s infidelity detection system evolved to overestimate the likelihood of their partner’s infidelity even more so than is the case for women. This idea is in line with Haselton and Buss’s (2000) error management theory; the costs of a compromised paternity by failing to detect cues of sexual infidelity may cause men to err by inferring infidelity even where none exists. As such, Andrews et al. (2008) documented that men were more likely than women to generate false positives than false negatives when making judgments about their partner’s past infidelities. Similarly, Goetz and Causey (2009) found that despite the fact that men were more likely than women to report that they themselves would commit sexual infidelity in the future, they were still more suspicious of their partner’s future infidelity than were women.

Despite these sex differences in suspicion, when it comes to intrasexual competition, both sexes view rivals with a high mate value as particularly threatening (Buss, Shackelford, Choe, Buunk, & Dijkstra, 2000), especially when the rivals have a more attractive body configuration (Buunk & Dijkstra, 2005; Dijkstra & Buunk, 2001) or a more attractive face (Massar & Buunk, 2010). This is even the case with voice; men with more masculine-sounding voices and women with more feminine-sounding voices elicit greater jealousy responses and are perceived more frequently to be intrasexual threats and rivals (O’Connor & Feinberg, 2012).

## Current Study

We examined whether it was possible to determine whether an individual had previously cheated on an exclusive, romantic partner by solely listening to that person’s voice. First, we expanded upon the procedures of O’Connor et al. (2011) by using nonmanipulated, natural voices, and compared listeners’ ratings for likelihood to cheat with reports of speakers’ actual history of infidelity. Because of the possibility that raters may assume that those with higher mate value were more likely to cheat simply due to increased sexual opportunity, we aimed to control for aspects of the voice that may contribute to the perception of a speaker’s mate value, particularly voice attractiveness and vocal pitch. Therefore, we selected voice samples from a larger pool of speakers obtained from a previous study (see Hughes et al., 2004) as our stimuli that differed between groups on self-reported cheating history but matched for several attributes related to mate value. Second, in an attempt to replicate the findings of O’Connor et al. (2011), we also manipulated the voice samples for pitch to see whether raters ascribed cheating behaviors more for the higher pitched female voices and lower pitched male voices, and we examined how this may relate to actual records of infidelity. However, evidence regarding the effect of a higher vocal pitch on perceptions of women’s attractiveness is not conclusive in the literature, with several studies demonstrating higher attractiveness ratings for lowered pitch female voices (Babel, McGuire, & King, 2014; Hughes et al., 2010, 2014). Because a lowered female voice may relate to sexual intent and/or arousal by the female speaker (Henton & Bladon, 1985; Hughes et al., 2010; Karpf, 2006), we predicted that women with lowered pitched voices may be evaluated as having a greater likelihood of committing infidelity.

## Method

### Participants

One hundred fifty-two undergraduate students (64 men and 88 women) from a small northeastern U.S. college served as independent raters for this study. Their mean age was 20.01 (*SD* = 2.13; age ranging from 18 to 32). Participants’ reported ethnicities were as follows: 65% Caucasian/White, 23% African-American/Black, 7% Hispanic/Latino, 4% Asian, and 1% other. Only those who reported having a heterosexual orientation were included in the analyses reported above. Additionally, 45% reported currently being in an exclusive, committed, romantic relationship, while 55% reported that they were not.

Participation in this study was voluntary, and all procedures were approved by the local institutional review board. Participants who were enrolled in psychology courses at the college were given the opportunity to receive extra credit as compensation for their participation, although some participants volunteered for no remuneration.

### Vocal Stimuli

We obtained voice samples from a larger data set (see Hughes et al., 2004) that were of a number count from 1 to 10, where speakers were instructed to speak at a pace of approximately one numeral per second. Following the procedures of O’Connor et al. (2011) who had used nine original male voices and nine original female voices, we selected 10 male voices and 10 female voices for this study. Half of the speakers for each sex reported that they had sexual intercourse with a person outside of a previous or current, exclusive and committed romantic relationship at some point in their lives (i.e., were “cheaters”), and the other half reported never cheating on their partners. All voice samples were of individuals who reported being heterosexual, White, unmarried, and were currently in a committed, exclusive relationship, thus assuring that speakers had at least one relationship opportunity to cheat in their lives.

In an attempt to control for aspects of the voice that may contribute to the perception of a speaker’s mate value, we selected voice samples of speakers who matched between the cheater and noncheater groups on criteria related to mate value. As shown in Table 1, there were no mean differences between the cheater and noncheater voice samples in terms of the speaker’s age, independent voice attractiveness ratings made on a 5-point rating scale (as described in Hughes et al., 2004), and voice pitch for each sex, all of which may affect one’s perception of a speaker’s mate value (Apicella & Feinberg, 2009; Hughes et al., 2004; Hughes & Rhodes, 2010).

**Table 1.** A Comparison of Speaker Characteristics Between Those Reporting Past Infidelity From Those Who Did Not.

		Male Voices			Female Voices		
Speaker traits	Speaker group	*M* (*SD*)	*t*	*p*	*M* (*SD*)	*t*	*p*
Age	Cheater	21.00 (1.23)	1.24	.249	19.60 (2.07)	−0.27	.792
	Noncheater	19.40 (2.61)			20.00 (2.55)		
Voice characteristics							
Voice attractiveness ratings	Cheater	3.39 (0.15)	0.77	.463	3.48 (0.34)	1.06	.319
	Noncheater	3.28 (0.30)			3.29 (0.21)		
Mean pitch	Cheater	137.19 Hz (16.36)	0.38	.718	194.62 Hz (8.50)	−1.13	.290
	Noncheater	133.14 Hz (17.82)			200.57 Hz (8.11)		
f_0_ SD	Cheater	31.87 Hz (11.15)	0.78	.458	20.84 Hz (9.90)	−0.75	.474
	Noncheater	25.83 Hz (13.25)			25.55 Hz (9.88)		
Duration	Cheater	7.46 s (1.56)	0.05	.965	7.44 s (0.55)	−0.38	.714
	Noncheater	7.43 s (0.67)			7.61 s (0.87)		
Jitter factor	Cheater	0.78 (1.39)	0.82	.435	−0.45 (0.31)	−0.16	.880
	Noncheater	0.10 (1.19)			−0.43 (0.19)		
Shimmer factor	Cheater	0.62 (1.22)	0.43	.682	−0.39 (0.25)	0.71	.498
	Noncheater	0.26 (1.45)			−0.49 (0.20)		
HNR	Cheater	8.79 dB (3.04)	−1.10	.302	12.92 dB (2.64)	−0.84	.423
	Noncheater	11.14 dB (3.67)			14.35 dB (2.71)		
Body characteristics							
Height	Cheater	72.70 in. (1.86)	2.06	.074	62.80 in (2.39)	−1.01	.344
	Noncheater	70.40 in. (1.67)			64.45 in (2.79)		
Weight	Cheater	179.80 lb (24.05)	1.72	.124	124.80 lb (13.37)	−0.90	.395
	Noncheater	156.60 lb (18.24)			138.60 lb (31.62)		
BMI	Cheater	23.66 (3.25)	1.32	.223	22.24 (1.88)	−0.58	.581
	Noncheater	21.48 (1.75)			23.30 (3.66)		
WHR	Cheater	0.90 (0.51)	1.43	.191	0.75 (0.08)	−0.16	.876
	Noncheater	0.81 (0.02)			0.76 (0.03)		
SHR	Cheater	1.17 (0.07)	0.86	.413	1.04 (0.09)	0.28	.787
	Noncheater	1.13 (0.05)			1.03 (0.11)		
Sexual behaviors							
Age of first masturbation	Cheater	12.60 (0.54)	−0.59	.572	14.00 (7.07)	0.30	.793
	Noncheater	13.00 (1.41)			12.50 (0.71)		
Age of first sex	Cheater	16.80 (1.30)	0.34	.741	16.40 (0.54)	−1.04	.334
	Noncheater	16.50 (1.29)			17.00 (1.15)		
Number of sex partners	Cheater	4.20 (1.30)	0.00	1.00	4.40 (2.19)	0.54	.603
	Noncheater	4.20 (5.17)			3.40 (3.51)		

*Note.* *p < .05. **p < .01.

Table 1 also presents a comparison between speaker groups for other acoustic measures that relate to the perception of voice quality such as fundamental frequency *SD*, jitter, shimmer, harmonics-to-noise ratio (HNR), and voice sample duration. Using Praat 5.3.70 software, five jitter measures were taken (local, local absolute, relative average perturbation, 5-point period perturbation quotient, and difference of differences of periods) and six shimmer measures were taken (local, local dB, 3-, 5-, and 11-point amplitude perturbation quotient, and difference of differences of periods). A principal component factor analysis was performed for the jitter and shimmer measurements separately, resulting in one jitter factor (eigenvalue: 4.823; Kaiser–Meyer–Olkin sampling adequacy: 0.850; Bartlett’s test of sphericity:χ^2^ = 304.12, *p* < .001) and one shimmer factor (eigenvalue: 5.911; Kaiser–Meyer–Olkin sampling adequacy: 0.891; Bartlett’s test of sphericity:χ^2^ = 510.06, *p* < .001). Factor regression scores were compared between cheater and noncheater groups for each sex and are reported in Table 1. Our acoustic measures are similar to other reports of voice measures in normal, young adults (Naufel de Felippe, Grillo, & Grechi, 2006; Toran & Lal, 2009).

Further, since previous investigations have shown that voice is related to particular body measures (see Hughes et al., 2004, 2009) and sexual behaviors (Hughes et al., 2004), we also selected speakers between the two groups who showed no difference in their height, weight, waist-to-hip ratio (WHR), shoulder-to-hip ratio (SHR), or body mass index (BMI), and reported measures of certain sexual behaviors (i.e., age of first masturbation, age of first sexual intercourse, number of sexual partners throughout lifetime) for each sex, as shown in Table 1.

### Pitch Manipulation

In addition to using natural voice samples, we created two versions of each voice recording, a raised pitch version, and a lowered pitch version, using Pratt 5.3.70 software. Following the procedures of O’Connor et al. (2011), voice pitch was manipulated by +0.50 ERB for a higher pitch and −0.50 ERB for a lower pitch from their baseline, allowing for the degree of pitch manipulation to be perceived equivalently regardless of the natural pitch. The manipulated pitches for female voices were significantly different from normal pitch (*M* = 197.60 Hz, *SD* = 8.44) for both raised pitch samples, *M* = 232.28 Hz, *SD* = 10.04; *t*(9) = 11.40, *p* < .001, and lowered pitched samples, *M* = 169.84 Hz, *SD* = 10.20; *t*(9) = 9.84, *p* < .001. Likewise, the manipulated pitches for male voices were significantly different from normal pitch (*M* = 135.17 Hz, *SD* = 16.27) for both raised pitch samples, *M* = 159.29 Hz, *SD* = 16.79; *t*(9) = 6.96, *p* < .001, and lowered pitched samples, *M* = 104.61 Hz, *SD* = 13.34; *t*(9) = 5.72, *p* < .001.

The pitch of the raised male voices was not significantly different between the cheater (*M* = 158.51 Hz, *SD* = 21.77) and noncheater groups (*M* = 160.07 Hz, *SD* = 12.61), *t*(8) = 0.14, *p* = .893, nor was the pitch for the lowered male voices for cheater (*M* = 102.91 Hz, *SD* = 13.78) and noncheater groups (*M* = 106.31 Hz, *SD* = 14.25), *t*(8) = 0.38, *p* = .712. Similarly, there was no difference in pitch between cheater (*M* = 230.00 Hz, *SD* = 8.91) and noncheater groups (*M* = 234.56 Hz, *SD* = 11.61) for the raised female voices, *t*(8) = 0.70, *p* = .505, or between cheater (*M* = 170.02 Hz, *SD* = 6.01) and noncheater groups (*M* = 169.66 Hz, *SD* = 14.07) for the lowered female voices, *t*(8) = 0.05, *p* = .960.

To assure that our manipulated samples sounded natural, prior to the study, we conducted a perceptual manipulation check using independent raters (*N* = 7). Each of these raters were asked to listen to each manipulated voice sample to determine whether the voice sounded like a natural voice or manipulated voice sample, and all raters (100%) confirmed that each manipulated voice had the quality and naturalness of a nonmanipulated voice.

### Independent Ratings

We tested participants either individually or in small groups in a private, quiet setting. After raters completed a brief demographic questionnaire, they answered a set of questions designed to determine the degree to which they thought engaging in certain sexual behaviors with others when in an exclusive committed, romantic relationship was considered an “act of cheating” on their partner. Respondents were asked to rate different sexual acts using a 10-point scale ranging from 1 = *not at all cheating* to 10 = *very much so cheating*. The five sexual acts rated were kissing on the lips, kissing with tongue, intimate touching/fondling, oral sex, and sexual intercourse. These questions were generated by the research team to first gain a sense of what respondents considered were acts of cheating since they were later asked to assess how likely they believed speakers had cheated on their partners. The interrater reliability was relatively high for these items (Cronbach’s α = .83).

Participants then listened to voice samples through stereophonic computer speakers and were asked, “Using the following scale, please rate how likely you think the person speaking has ‘cheated’ on their romantic partner with whom they are in an exclusive, committed relationship.” They gave responses for each voice on a 10-point scale ranging from 1 = *not at all likely* to 10 = *very likely*. In contrast to the forced-choice paradigm that O’Connor et al. (2011) used, we obtained interval scale measures to allow for greater variability in ratings and for parametric analyses.

To avoid fatigue effects, we did not ask raters to judge all 60 voice ratings (20 natural and 40 manipulated voices samples). Rather, each participant rated 20 voice samples. The 20 natural voice samples were rated by 54 participants (25 men and 29 women). The manipulated female voices were rated by another set of 45 participants (19 men and 26 women) while the manipulated male voice samples were rated by another 53 participants (20 men and 33 women). Voice samples were played by the experimenter in a counterbalanced order. The interrater reliability was adequate for both the normal voice samples (Cronbach’s α = .71) and for the manipulated pitch samples (Cronbach’s α = .73).

## Results

### Assessment of “Acts of Cheating”

We conducted a 5(type of sexual act) × 2(respondent sex) mixed-model analysis of variance (ANOVA) to examine the extent to which respondents thought a given sexual behavior was considered an “act of cheating” on a partner when in an exclusive, committed, romantic relationship. There was a main effect for sexual act, *F*(4, 496) = 36.73, *p* < .001, η^2^ = .228, and post hoc analysis revealed that all pairwise comparisons were significantly different from one another with the exception of touching/fondling and kissing with tongue (presented in the order of magnitude: sexual intercourse, *M* = 9.89, *SE* = 0.07; oral sex, *M* = 9.76, *SE* = 0.09; intimate touching/fondling, *M* = 9.07, *SE* = 0.14; kissing with tongue, *M* = 9.03, *SE* = 0.16; and kissing on the lips, *M* = 8.33, *SE* = 0.22; *p* values < .001). There was no main effect for the sex of the respondent, *F*(1, 124) = 1.51, *p* = .222, η^2^ = .012, nor a significant interaction between respondent sex and sexual act, *F*(4, 496) = 0.82, *p* = .511, η^2^ = .007.

### Cheating Assessment: Natural Voices

A binary logistic regression was performed to determine which of the acoustic measures (mean pitch, F_0_*SD*, jitter factor, shimmer factor, HNR, duration) along with speaker sex were predictors of the whether a speaker’s voice was that of a cheater or not. The overall logistic regression model was not reliable in distinguishing between the voices of cheaters and noncheaters, χ^2^(7) = 8.15, *p* = .320; Nagelkerke *R*^2^ = .446, −2 log likelihood = 19.58, and none of the variables in the equation added significantly to the model.

We used a 2(cheater/noncheater group) × 2(speaker sex) × 2(rater sex) mixed-model ANOVA to analyze mean ratings of how likely participants thought the speakers had cheated on a romantic partner while in an exclusive, committed relationship for the nonmanipulated voice samples. The within-subject factors were the speaker cheater group and the speaker sex, as these voice samples were heard by each rater, while the rater sex was the between-subject factor. Dependent measures were calculated as mean values for each of the four vocal conditions heard by each rater: (1) male voices/cheater group, (2) male voices/noncheater group, (3) female voices/cheater group, and (4) female voices/noncheater group. Preliminary assumptions were performed to check for normality, linearity, outliers, and homogeneity of variance with no significant issues noted. There was a main effect of cheater versus noncheater group, whereby raters thought speakers who reported that they had cheated on a romantic partner were more likely to be suspected as being a cheater (*M* = 5.02, *SE* = 0.13) than speakers who reported not to have cheated on romantic partners (*M* = 4.59, *SE* = 0.11), *F*(1, 52) = 11.95, *p* = .001, η^2^ = .187. There was also a main effect for the sex of the speakers, *F*(1, 52) = 27.67, *p* < .001, η^2^ = .347; overall, male speakers were rated as more likely to have cheated (*M* = 5.14, *SE* = 0.13) than were female speakers (*M* = 4.47, *SE* = 0.11). Likewise, there was a main effect for the sex of the raters, *F*(1, 52) = 7.03, *p* = .011, η^2^ = .119, whereby female raters gave higher ratings for cheating (*M* = 5.08, *SE* = 0.14) than had male raters (*M* = 4.53, *SE* = 0.15). There were no significant interactions found (see Table 2 for descriptive statistics).

**Table 2.** Descriptive Statistics of Infidelity Ratings.

		Male Voices		Female Voices	
Voice Sample	Speaker Group	*M*	*SD*	*M*	*SD*
Natural voice	Cheater	5.35*	1.22	4.73	1.04
	Noncheater	4.99	1.10	4.24***	1.03
Raised pitch	Cheater	4.71*	1.03	4.75	1.25
	Noncheater	4.32***	1.00	4.21***	1.31
Lowered pitch	Cheater	5.28	1.09	5.56*	1.50
	Noncheater	5.05	1.29	4.88	1.07

*Note*. *p < .05. **p < .01. and ***p < .001 indicate whether mean rating made on 10-point scale is significantly different from the midpoint value of 5 (chance).

To account for any effects of ethnicity, we considered the mean ratings made only by raters who were of the same ethnicity as all the speakers (White, *N* = 40, 23 men). The main effect for cheater group, *F*(1, 38) = 18.57, *p* < .001, η^2^ = .328, and main effect for sex of speaker, *F*(1, 38) = 20.85, *p* < .001, η^2^ = .354, remained; however, there was no main effect for sex of rater, *F*(1, 38) = 2.11, *p* = .154, η^2^ = .053. Both the age and relationship status of the raters did not affect the findings.

### Cheating Assessment: Manipulated Voices

A 2(cheater/noncheater group) × 3(lower/higher pitch) × 2(rater sex) factorial analysis was used to examine whether the manipulated pitch voice samples influenced ratings of the likelihood speakers were thought to have cheated on their partners. Because the manipulated voices of male and female speakers were judged by different raters, we analyzed male and female voices separately (see Table 2 for descriptive statistics). For manipulated male voices, the main effect of cheater versus noncheater group remained evident, whereby raters thought male speakers who reported that they had cheated on a romantic partner were more likely to be suspected as being a cheater (*M* = 4.94, *SE* = 0.13) than speakers who reported not to have cheated on their romantic partners (*M* = 4.61, *SE* = 0.12), *F*(1, 51) = 6.93, *p* = .011, η^2^ = .120. There was also a main effect for voice pitch whereby manipulated lower pitched male voices (*M* = 5.08, *SE* = 0.15) were given higher cheating ratings than manipulated higher pitched male voices (*M* = 4.47, *SE* = 0.12), *F*(1, 51) = 14.01, *p* < .001, η^2^ = .216. Further, there was a main effect for the sex of the raters, *F*(1, 51) = 5.51, *p* = .023, η^2^ = .097, whereby female raters gave speakers of manipulated male voices higher ratings for cheating (*M* = 5.03, *SE* = 0.13) than had male raters (*M* = 4.52, *SE* = 0.17). There were no significant interactions found for the manipulated male voices.

For manipulated female voices, the main effect of cheater versus noncheater group also remained, whereby raters thought female speakers who reported that they had cheated on a romantic partner were more likely to be suspected as being a cheater (*M* = 5.11, *SE* = 0.15) than speakers who reported not to have cheated (*M* = 4.56, *SE* = 0.14), *F*(1, 43) = 12.52, *p* = .001, η^2^ = .225. There was also a main effect for voice pitch whereby manipulated lower pitched female voices (*M* = 5.20, *SE* = 0.16) received higher cheating scores than manipulated higher pitched voices (*M* = 4.48, *SE* = 0.18), *F*(1, 43) = 9.05, *p* = .004, η^2^ = .174. Unlike for male manipulated voices, there was no main effect for sex of rater for female manipulated voices, *F*(1, 43) = 0.53, *p* = .472, η^2^ = .012. In addition, there was a significant three-way interaction between voice cheater group, manipulated pitch, and rater sex, *F*(1, 43) = 10.76, *p* = .002, η^2^ = .200. When divided by rater sex, male raters were able to distinguish between manipulated higher pitched female voices of women who had reported cheating (*M* = 4.68, *SD* = 1.51) from women who had not (*M* = 4.17, *SD* = 1.45), *t*(18) = 2.42, *p* = .026, Cohen’s *d* = .344. However, male raters did not rate female voices with a manipulated lower pitch any differently based upon whether the speaker had a history of cheating (*M* = 5.04, *SD* = 1.02) or not (*M* = 5.11, *SD* = 1.15), *t*(18) = 0.21, *p* = .833. On the other hand, female raters found female speakers who reported having cheated on a romantic partner as more likely to have cheated regardless if their pitch was manipulated to sound higher (cheater: *M* = 4.80, *SD =* 1.05; noncheater: *M* = 4.25, *SD* = 1.23, *t*(25) = 2.39, *p* = .025, Cohen’s *d* = .481) or lower (cheater: *M* = 5.93, *SD* = 1.69; noncheater: *M* = 4.72, *SD* = 0.99, *t*(25) = 4.56, *p* < .001, Cohen’s *d* = .873).

To examine whether listeners were performing above or below chance levels, several single-sample *t*-tests were conducted and reported in Table 2. Only natural voices of male cheaters and manipulated, lowered voices of female cheaters had mean infidelity ratings that exceeded the chance value of 5 on the 10-point rating scales. Natural voices of female noncheaters, manipulated, raised voices of male cheaters and noncheaters, and manipulated, raised voices of female noncheaters had infidelity ratings that fell significantly below chance value.

## Discussion

### What Is Cheating?

We first wanted to gain a better understanding of which sexual acts our raters agreed had constituted cheating behaviors since they were later asked to rate how likely a speaker was a cheater. We found that although most behaviors (even fondling, touching, and kissing) were rated high on the scale as acts considered to be cheating, participants thought sexual intercourse and oral sex were the most likely behaviors to signify cheating. These findings are in line with more comprehensive studies that have documented which sexual and erotic acts are perceived as cheating behaviors (Kruger et al., 2013; Yarab, Sensibaugh, & Allgeier, 1998). It is also worth noting that there were no sex differences in defining which behaviors represented cheating. Similarly, Kruger et al. found that men and women were in agreement as to which sexual or erotic acts are deemed cheating behaviors. However, these researchers addressed the possibility that the data were limited by a ceiling effect, since both men and women rated most acts as being highly indicative of cheating, as was the case in our study. Needless to say, the identification of “cheating” behaviors was operationalized by our participants and revealed that engaging in a variety of sexual acts was considered “cheating.”

### Voice and Cheater Detection

We found that individuals can use cues in a voice to determine whether a speaker had cheated on their romantic partners. By simply hearing a brief voice recording, raters judged actual cheaters as more likely to have cheated. These data provide further evidence that the human voice can be of value as a cheater detection tool. Since we controlled for voice attractiveness, voice pitch and other acoustic measures, and a variety of features related to mate value (e.g., age, BMI, WHR, SHR, certain sexual behaviors), this effect was not dependent upon obvious vocal cues that may signal a speaker’s higher mate value or on features to suggest the person may have had more sexual opportunity to have affairs.

These findings also extend those of O’Connor et al. (2011) by showing that the sound of one’s voice can be used as a medium to assess a person’s reported past history of infidelity. While O’Connor and colleagues’ study showed how manipulating pitch plays a role in appraising a speaker’s proclivity to be unfaithful, our study demonstrated that perceptions of a speaker’s actual history of infidelity could, in fact, be determined through voice alone. Further, these findings demonstrate the validity of Ambady and Rosenthal’s (1992) thin slice theory, whereby one can make accurate assessments of another based on merely brief observations. These data also suggest that cognitive strategies have evolved to facilitate wise mate choices, holding true even when only the auditory sensory modality is operating.

We were unable to identify exactly which acoustic qualities were driving the perception of cheating ascriptions, albeit a detailed acoustical analysis beyond the aims and scope of this perceptual study. It is interesting, then, to speculate what aspects of the human voice raters were using to make these accurate assessments because we eliminated differences between groups for the more conspicuous cues of a voice that could be driving factors (i.e., variations in vocal attractiveness, voice pitch, and other basic acoustic features). While evidence shows that a voice can be used to assess various traits and behaviors, the acoustic parameters that allow for these various voice assessments are largely unknown (Hughes et al., 2008). There is also considerable debate about which exact properties of a voice allow for the perception of assessing an attractive-sounding voice (see Hughes & Gallup, 2008), and it may be a unique constellation of different acoustic features that contribute to perceived vocal attractiveness (Babel et al., 2014).

There could be a host of possible vocal parameters beyond those measured in this study that may be responsible for “leaking” information about one’s history of infidelity, as demonstrated from studies examining vocal emotion expression (see Patel, Scherer, Björkner, Sundberg, 2011). Other vocal cues such as clarity of articulation may have also contributed to perceptions of infidelity. For example, masculine men tend to display less clarity in their speech and show phonetic patterns indicative of masculinity, which in turn could be associated with infidelity threat (Kempe, Puts, & Cárdenas, 2013). However, as mentioned, a more detailed acoustic analysis of our voice samples was beyond the scope of this perceptual study and would have required a far larger sample size of vocal stimuli to allow one to appropriately interpret such analyses. Further, because we used stimuli that were not intended for a more detailed acoustic analysis (i.e., we did not have speakers provide sustained vowel utterances), we were limited in the features we could accurately assess (Parsa & Jamieson, 2001). As Parsa and Jamieson noted, analysis of continuous speech can be more challenging than examining sustained vowels because of the inherent inconsistency of the signal and unvoiced silent regions of speech.

It may be the case that appraisal for the “propensity for infidelity” is a reflection of personality traits that can be revealed through the voice (Mairesse, Walker, Mehl, & Moore, 2007). For instance, extroverts show greater variation in fundamental frequency, greater voice quality, and fewer silent pauses (see Mairesse et al., 2007 for review), and high extroversion strongly predicts infidelity (Nettle, 2005; Nettle & Clegg, 2008). While our sample of voices was too small to appropriately make this sort of determination, it would be interesting to see whether particular speech patterns exist for those who have been identified via voice as having a greater proclivity to cheat on their partners and if this relates to particular personality measures.

It could be argued that these results are a reflection of past experience, and participants may have generalized attributes of voices of previous partners they had known to have cheated in order to make these assessments. While there is evidence that one can learn to better uncover deception and improve lie detection accuracy through vocal cues, the effect only holds true for decoding practice with a particular target, and it does not generalize to accuracy in detecting lies enacted by other deceivers (Zuckerman, Koestner, & Alton, 1984). A high level of accuracy when detecting deceit through voice is also thought to occur when spoken deceptions involve emotion (Ekman, O’Sullivan, Friesen, & Scherer, 1991). However, it is important to realize that the voice samples used in this study were not capturing a transient state of intentional deceit (as we had removed any content and emotion from the vocal samples by using a number count) but were revealing of more enduring trait aspects of the voice.

### Sex Differences in Cheating Detection

These findings also extend reports of sex differences in judging a partner’s faithfulness. Whereas the female raters in this study were not necessarily more accurate in ascribing whether a male speaker had actually cheated, they were more likely to suspect that male speakers had cheated than did the male raters assessing female speakers, and this held true for both natural and for manipulated male voices. This finding corroborates reports that both male and female raters tend to think that men are more likely to have cheated in their past (DeVries & Ajzen, 1971) and a large questionnaire study found that college women were much more likely to believe that all men cheat on a partner at least once (Knox, Zusman, & McNeely, 2004). In addition, we found that among the natural voices, male cheaters were the only group whose mean infidelity ratings exceeded chance level, while female voices of noncheaters fell below chance level. Perhaps this sex difference can be explained by reports that men are more likely to commit infidelity (Michael, Gagnon, Laumann, & Kolata, 1995) and adultery (Kinsey, Pomeroy, Martin, & Gebhard, 1953). Data from more recent studies also substantiate the claim that men are more likely to engage in extradyadic sexual activity than are women (Allen & Baucom, 2004) and men were more likely than women to report that they themselves would commit sexual infidelity in the future (Goetz & Causey, 2009).

When it comes to sex differences in cheater detection, Ein-Dor, Perry-Paldi, Hirschberger, Birnbaum, and Deutsch (2015) showed that women displayed greater alertness to cues of a potential partner unfaithfulness than do men; however, while women were quicker and more accurate in detecting cues of infidelity, they were not better at detecting actual threats from others. Further, Rhodes et al. (2013) found that women could accurately judge a man’s past infidelity simply from minimal facial cues and used masculinity as the valid cue to do so, whereas men were comparably less adept at this task. Andrews et al. (2008) also found sex differences in making inferences about a romantic partner’s infidelity, but in their study, men made more accurate inferences than women, as men’s ratio of positive to negative errors was greater. These findings are consistent with the error management theory which posits that, for men, the costs of failed pursuits (false positive errors) outweigh the costs of missed sexual opportunities (false-negative errors; Haselton & Buss, 2000). More research is needed to elucidate sex differences in infidelity detection.

### Voice Pitch and Cheater Detection

When manipulating pitch to determine its impact on perceptions of speakers’ infidelity, the effect of correct cheater identification remained despite these changes in pitch; participants were still attributing higher cheating ratings to those who had reported a history of cheating regardless of the manipulations in pitch. The one exception was that male raters could not distinguish between cheaters and noncheaters for women with lower pitch voices.

We replicated O’Connor et al.’s (2011) finding that men with manipulated lower pitched voices were more likely to be ascribed as cheaters. Similarly, a subsequent study conducted by O’Connor and Barclay (2017) also showed that listeners perceived higher pitched male voices as being more trustworthy, less likely to commit infidelity, and less likely to poach another’s romantic partner than lower pitched male voices. O’Connor, Pisanski, Tigue, Fraccaro, and Feinberg (2014) found that the more likely a woman believed that men with lower pitch voices engage in infidelity, the more likely she preferred men with low-pitch voices for short term as compared to long-term relationships. The authors suggested that this mechanism allows women to assess fitness and abandonment costs associated with infidelity.

Similar to O’Connor and Barclay (2017), we were also unable to replicate the findings of O’Connor et al. (2011) with regard to perceptions of infidelity relating to women with voices manipulated to be a higher pitch. Just as with male voices, we documented that women with voices manipulated to have a lower pitch were also thought to be more likely to have cheated than those with higher pitches. In fact, female cheaters whose voices were lowered were the only group of manipulated voices whose mean infidelity ratings exceeded chance level. O’Connor and colleagues (2011) attributed their findings to that fact that higher pitched female voices reflect higher estrogen and mate value, and therefore these women may have more opportunity to cheat. However, our findings suggest that perhaps testosterone, which is linked to both lower pitch (Abitbol et al., 1999; Damrose, 2009; Evans et al., 2008; Grisa et al., 2012) and sex drive, even in women (Abitbol et al., 1999; Davis, 2000), is what may be driving this perception for both sexes. Further, our findings are in line with evidence showing that women tend to speak with deeper voices when conveying sexual interest to men (Hughes et al., 2010; Karpf, 2006). In fact, when participants were asked to intentionally display a “sexy voice,” women had decreased the pitch of their voices even more than had men and showed greater success in being perceived as having a sexier voice (Hughes et al., 2014; Tuomi, & Fisher, 1979). Therefore, it is possible that raters associated a lower pitched female voice to women who were intentionally seeking casual sexual encounters such as affairs. In this same vein, it has been shown that female voices with a breathier voice quality were rated as more attractive (Babel et al., 2014). Henton and Bladon (1985) suggested that when a woman displays a breathier voice, she is imitating a voice quality associated with arousal, and if a woman can manage to *sound* as though she is sexually aroused, she may be regarded as more desirable by men. In our study, manipulated lower pitched female voices appeared to have made actual cheater detection for men more difficult; men were unable to decipher which speakers were cheaters for the lower pitch voices but were able to do so for the higher pitch female voices.

Another possible reason why our findings for manipulated female voices did not confirm those of O’Connor et al. (2011) may be methodological differences. O’Connor et al. used a two-alternative, forced-choice paradigm to select which speaker was more likely to cheat, whereas our study presented voice samples individually and used interval rating scales to generate more variability in responses. Our study also utilized longer vocal samples (i.e., a number count) as stimuli while O’Connor et al. used sustained vowel sounds. Studies examining how stimulus type affect voice attractiveness ratings have shown that word length samples tend to be rated as more attractive to the opposite sex than vowel sounds taken from the same targets (Ferdenzi et al., 2013), so perhaps the type of stimuli used in each study examining the detection of infidelity from voice captured different percepts. Nonetheless, O’Connor and Barclay (2017) were also unable to replicate the findings of O’Connor et al. (2011) by failing to demonstrate that female voice pitch had a significant effect on perceptions of infidelity. Because the authors used similar stimuli and degree of pitch manipulations as the previous investigation, they concluded that their failure to replicate the findings of O’Connor et al. (2011) was unlikely due to methodological differences between the two studies. Thus, additional work on how pitch contributes to the perception of infidelity of female voices is warranted.

### Limitations and Future Directions

There were limitations to this study. Our data regarding infidelity were based on self-report and may not have accurately depicted participants’ histories (i.e., because of social desirability, participants may have underreported their EPC history). It is also possible that given our young sample of speakers, there may have been less opportunity to cheat than in an older sample. In addition, we did not examine the cheating status of raters. Future studies could account for whether raters’ own history of infidelity plays a role in their ability to determine another’s infidelity, and this may better parse apart whether cheater detection aptitude through voice is more of an inherent or experiential ability. Moreover, it would be interesting to replicate these findings with those of different sexual orientations.

Because this was a perceptual study, we wanted to use continuous speech as our stimuli since this is a better representation of how perceptual judgments occur in “the real world” and would allow for greater ecological validity. Unlike examining sustained vowels which have been used in other perceptual studies, continuous speech can account for rapid voice onset and termination, variations in voice fundamental frequency and amplitude, and voice breaks (Parsa & Jamieson, 2001). Nonetheless, use of continuous speech stimuli comes at a cost in terms of acoustic analyses. When individual acoustic measures are considered in isolation, it has been shown that classification of voice quality is more accurate for measures derived from sustained vowels than for continuous speech samples (Parsa & Jamieson, 2001). In particular, perturbation measures such as shimmer and jitter extracted from continuous speech samples are likely to be influenced by intonation and other modulation effects and reduce the identification of voice quality, thus making it more challenging to examine objectively because of its dynamic signal.

Future work on this topic could also manipulate other features of the voice to assess what acoustic components are used to make this assessment outside of voice pitch. For instance, future investigations that utilize larger sample sizes of vocal stimuli could take into account how certain indexical features of speech may b**e** revealing of personal information about the speakers. As an example, both men and women judge a breathier female voice to be more attractive, and this appears to indicate healthier, younger, more feminine larynges (Babel et al., 2014). Future studies may wish to manipulate breathiness to determine effects on cheating ascriptions.

If pitch is a salient signal that impacts infidelity perceptions, matching pitch between cheaters and noncheaters may have obscured performance accuracy. It is possible that judgment would be influenced and even improved by allowing raters to hear naturally occurring pitch differences between speakers. Therefore, future investigations could also examine naturally occurring voices that are not matched in pitch to see if their accuracy to detect infidelity improves further with pitch variation.

## Conclusion

In sum, this study provides further evidence that voice may be used as a cue of mate infidelity. We were able to demonstrate that is it possible to determine whether a person had cheated on their romantic partners from hearing their voice alone. By using natural voice samples (i.e., those that were not experimentally manipulated) and self-reports of an actual history of infidelity, we were able to extend the external validity of previous investigations (O’Connor, Re, & Feinberg, 2011) by showing how voice can be used to uncover a speaker’s actual history of infidelity. While we cannot exactly pinpoint all the features about a voice that our perceptual system is using to make this assessment, we know that pitch plays a role, but does not represent the entire picture. We were able to replicate previous findings showing that pitch seems to be of importance when making this assessment, albeit not a necessary component since raters were still able to identify cheaters regardless of pitch manipulation (with the one exception of men’s assessment of lower pitch female voices). In addition to the many other cheater detection mechanisms we hold in our mating strategy repertoire, using voice to detect infidelity may have important implications for our mating success as seen from an evolutionary standpoint.
